# Variation characteristics of soil erosion and their response to landscape patterns in a typical basin in the Upper Yangtze River

**DOI:** 10.3389/fpls.2025.1523891

**Published:** 2025-03-20

**Authors:** Kaixin Jiang, Shuhong Mo, Jingzhe Zhang, Kunxia Yu, Zhanbin Li

**Affiliations:** ^1^ State Key Laboratory of Water Engineering Ecology and Environment in Arid Area Xi’an University of Technology, Xi’an, China; ^2^ Shaanxi Geology Mining 908 Environmental Geology Co., Ltd., Shaanxi Geology and Mining Group Co., Ltd., Xi’an, China

**Keywords:** Upper Yangtze River, soil erosion, geographical detector, land use, landscape pattern

## Abstract

Soil erosion is undeniably a significant cause of a variety of problems in the Upper Yangtze River (UYR), including floods, land degradation, and sedimentation in rivers. Recognizing alterations in soil erosion and its influencing variables in this area recently is a crucial scientific challenge requiring prompt solutions in regional soil erosion control. This study examines soil erosion and its influencing factors in the Jialing River Basin (JRB) from 1990 to 2018 using RUSLE and geographical detector. It focuses on the relationship between land use, landscape patterns, and soil erosion in this typical basin in the UYR. The results indicated that: (1) The average soil erosion modulus of the JRB decreased during 1990-2018, with predominant slight (< 500 t·km^−2^·a^−1^) and light (< 2,500 t·km^−2^·a^−1^) erosion intensity. Moderate and higher grades of erosion mainly occurred in the middle and lower JRB. (2) Cultivated land, forest land, and grassland accounted for over 97% of the JRB’s land use from 1990-2018, with cultivated land dominating the middle and lower areas. Over the years, there was an increase in forest land and construction areas, while cultivated land decreased. The landscape pattern was characterized by diversity, fragmentation, and decentralization. (3) The soil erosion control area (SECA), primarily situated in the middle and lower JRB, was predominantly cultivated land. Between 1990 and 2018, the SECA area underwent significant changes, with the most notable changes occurring in the lower Fujiang River Basin (FRB) and the western and middle parts of the Qujiang River Basin (QRB). The area experienced more fluctuations on the left bank of the JRB and the right bank of the JRB, specifically in the QRB and FRB. The research can serve as a reference for future decision-making on land use planning and soil erosion management in the UYR.

## 1 Introduction

Soil erosion, a phenomenon closely linked to the delicate balance of Earth’s ecosystems, poses a significant threat not only to global soil degradation but also to our water and marine resources (Borrelli et al., 2020; Prăvălie et al., 2021). As a country that once experienced severe erosion on a global scale, China has made significant progress in combating severe soil erosion through persistent ecological governance (Cao et al., 2023; Qiao et al., 2024). The large rivers in China have significantly reduced water and sediment since the 1950s. From 2000 to the present, the Yangtze River Basin (YRB) has overtaken the Yellow River, which was once renowned for its immense sediment concentration and unparalleled sediment transport in the world at one time, and has developed into an important marine sediment transport river (Yin et al., 2023; Zhang et al., 2023). Thus, elucidating the characteristics and reasons behind the current variations in soil and water loss in the YRB would help to scientifically optimize the layout and implementation of ecological and environmental protection measures. This, in turn, will promote the social economy in the basin to expand at a high standard.

As an empirical model that can effectively estimate soil erosion modulus (SEM), RUSLE and Chinese Soil Loss Equation (CLSE) (Liu, 2024) have been used by many scholars to analyze the spatio-temporal variation (Pinson and Aubuchon, 2023; Zhang et al., 2021a), driving factors (Peng et al., 2022) and other issues. Borrelli et al. (2017) quantitatively evaluated soil erosion on a global scale based on RUSLE, and assessed the impact of land use after 2000. Wuepper et al. (2019) utilized RUSLE to evaluate SEM in different countries and explored the correlation between SEM and various nations. Tang et al. (2023b) evaluated the soil erosion situation of the Northeast Plain of China, which is the third black soil region worldwide (Cao et al., 2024), based on RUSLE and quantitatively analyzed that the primary driving factor is human activity. Relying on RUSLE, Li et al. (2022b) investigated the erosion status of the Loess Plateau, an ecologically fragile region worldwide, since 1901 and found that the erosion rate was mainly driven by land management. In the Zhifanggou watershed, a representative small watershed on the Loess Plateau, Ma et al. (2024) quantitatively evaluated the impact of vegetation change and terrace construction on soil erosion using CLSE. Wang et al. (2020) evaluated soil erosion intensity in the red soil hilly region of southern China using CLSE and identified socioeconomic factors affecting soil erosion. Gao et al. (2023) analyzed spatio-temporal variations in erosion in Changting County, a representative area of the red soil region, based on RUSLE. Currently, the research areas of related studies are mostly large-scale or small-scale, with most concentrated in Yellow River and Loess Plateau. Studies on the Upper Yangtze River (UYR) with significant soil erosion are comparatively rare. In the same region, climate, soil, topography and geomorphology remain largely unchanged in the short term, but land use has been shown to significantly affect erosion (Chen et al., 2023b). However, current research tends to analyze land use types but lacks analysis of landscape patterns. Additionally, the analysis method is relatively simple. This study introduces the geographical detector, a statistical approach that can identify spatial differences and uncover the underlying driving factors without requiring a linear hypothesis assumption (Wang and Xu, 2017) and has been recently used in erosion research (Chen et al., 2024; Li et al., 2022a).

Within the YRB, the UYR stands out as a critically important region, renowned for its high potential for soil erosion and significant sediment yield (Liu et al., 2023b; Wang et al., 2022a). Recognizing the region’s vulnerability, the Chinese government has taken a proactive stance on environmental conservation, approving numerous key prevention and control projects specifically designed to tackle the challenges of soil and water conservation in the UYR. This official endorsement and subsequent implementation of various environmental protection initiatives in the region began in 1989, spurred on by the government’s commitment to sustainable development and the need to preserve the YRB’s delicate ecosystem. The Jialing River Basin (JRB), occupying a substantial area within the UYR, is renowned for its significant role as a major sediment-producing region in the region and is recognized as the primary sediment source for the renowned Three Gorges Reservoir Area (Liu et al., 2022b). This study focuses on the JRB, and undertakes a comprehensive analysis of spatio-temporal variation characteristics of erosion from 1990 to 2018 using two key methodologies: the RUSLE and the barycenter migration model. These models help in the detailed computation and visualization of erosion patterns over this period. We also utilize a geographical detector to identify the driving factors behind observed erosion patterns. The study then examines the relationship between landscape pattern indices and soil erosion, analyzing data to reveal direct and indirect correlations between various landscape features and soil loss. The study culminates in the generation of a soil erosion control area (SECA) for the basin, which presents insights to guide decision-making processes related to soil erosion control and land use planning within the UYR, with practical applications in the realm of land management and policy formulation.

## 2 Materials and methods

### 2.1 Study area

**Figure 1:**
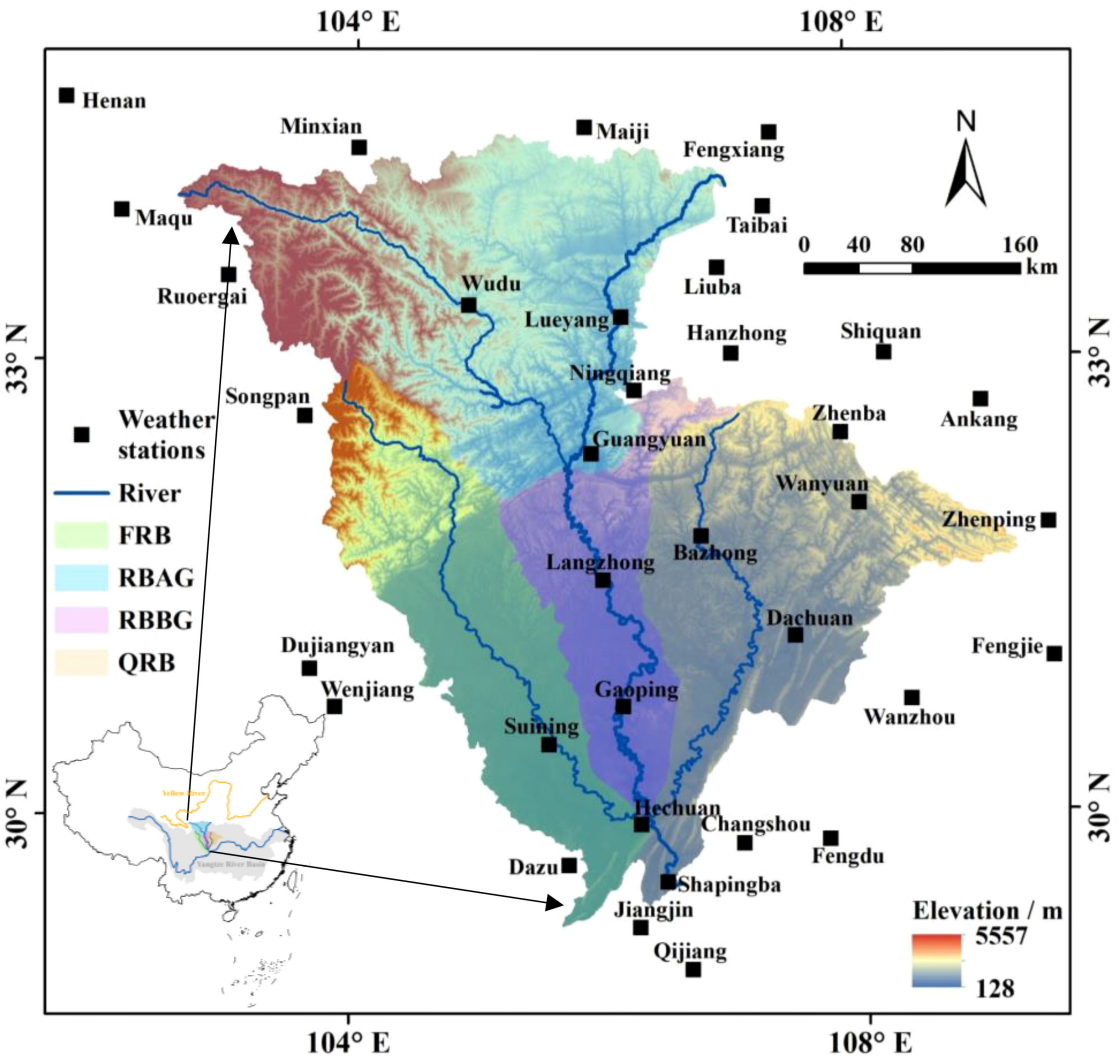
Location of the JRB.

With an area of over 160,000 km^2^, the JRB is the largest river in the YRB water system and the main left-bank tributary of the UYR in China (**Figure 1**). The river originates from the Qinling Mountains at the watershed between the YRB and Yellow River Basin. The area surrounding the basin extends from the northwest and rotates clockwise to the southeast, encompassing mountain ranges such as Qinling, Daba, Huaying, and Longmen. The middle and lower JRB predominantly encompass the hilly area of Sichuan, which is one of the three major geographical units that make up the Sichuan Basin. The Mesozoic purplish red sandstone and mudstone in this area are brittle and vulnerable to erosion and weathering. The middle and lower JRB are prone to serious flood disasters, influenced by the Daba Mountain rainstorm area and the rainstorm area of the Longmen Mountains. Additionally, the densely populated area with developed agriculture has caused significant soil erosion. The sediment concentration in the JRB is the highest among all water systems in the YRB, categorizing it as a key sediment-producing area in the UYR (Changjiang Water Resources Commission of the Ministry of Resources, 2018). The basin’s extensive fan-shaped water system can be divided into three major water systems: Fujiang River Basin (FRB), JRB trunk, and Qujiang River Basin (QRB). Furthermore, the trunk can be further divided into the river basin above Guangyuan (RBAG) and below Guangyuan (RBBG).

### 2.2 Study data

The study area’s DEM is derived from ASTER GDEM, a 30m resolution digital elevation data product obtained from the Geospatial Data Cloud (https://www.gscloud.cn/). To complement our analysis of land use, we extracted data from the Resource and Environmental Science Data Platform of the Chinese Academy of Sciences (https://www.resdc.cn), which provided us with land use data spanning from 1990 to 2018 and vegetation index data, both at a resolution of 30m. Furthermore, we tapped into the China surface climate daily data set (V3.0), accessible on the China Meteorological Data Network (http://data.cma.cn), to derive critical precipitation data. Finally, we obtained soil data from the Harmonized World Soil Database (https://gaez.fao.org/pages/hwsd).

### 2.3 Methodology

#### 2.3.1 RUSLE model

Soil erosion refers to the processes associated with the destruction and transportation of the physical structure or chemical composition of soil due to external forces like water, wind, freeze-thaw, or gravity (Hao et al., 2023). The RUSLE model, operating on the foundational principle of soil erosion equilibrium, is a widely used and well-regarded model for performing quantitative soil erosion assessments. Renowned for its combination of good accuracy and user-friendly ease of use, the model has become a popular tool in both soil conservation and management (Formula 1). As follows:


(1) E=R·K·L·S·C·P

(2) $$R_{i}=\alpha {\sum_{j=1}^{k}\left(P_{j}\right)}^{\beta }$$

(3) $$\beta =0.8363+18.144/P_{\left(d12\right)}+24.455/P_{\left(y12\right)}$$

(4) $$\alpha =21.586\beta^{-7.1987}$$

(5) $$K=0.1317\times \left(-0.01383+0.51575K_{EPIC}\right)$$

(6) $$\begin{array}{c} K_{EPIC}=\left[0.2+0.3e^{-0.0256m_{s}\left(1-\frac{m_{silt}}{100}\right)}\right]\\ \cdot {\left(\frac{m_{silt}}{m_{c}+m_{silt}}\right)}^{0.3}\cdot \left(1-\frac{0.25orgC}{orgC+e^{3.72-2.95C}}\right)\\ \cdot \left(1-\frac{0.7SN1}{SN1+e^{-5.51+22.9SN1}}\right) \end{array}$$

(7) $$SN1=1-\frac{m_{s}}{100}$$

(8) $$L={\left(\frac{\lambda }{22.13}\right)}^{m},\;m=\left\{ \begin{array}{l} 0.2,\;\theta \leq 1^{\circ }\\ 0.3,\;1^{\circ }<\theta \leq 3^{\circ }\\ 0.4,\;3^{\circ }<\theta \leq 5^{\circ }\\ 0.5,\;\theta >5^{\circ } \end{array}\right.$$

(9) $$S=\left\{ \begin{array}{l} 10.8\sin\theta +0.03,\;\theta \leq 5^{\circ }\\ 16.8\sin\theta -0.5,\;5^{\circ }<\theta \leq 10^{\circ }\\ 21.9\sin\theta -0.96,\;\theta >10^{\circ } \end{array}\right.$$

(10) $$C=\left\{ \begin{array}{l} 1,\;0\leq FVC\leq 0.001\\ 0.6508-0.3436\log FVC,\;0.001<FVC\leq 0.783\\ 0,\;FVC>0.783 \end{array}\right.$$

(11) $$FVC=\frac{NDVI-NDVI_{\min}}{NDVI_{\max}-NDVI_{\min}}$$

where *E* is SEM (t·km^–2^·a^–1^), *R* is rainfall erosivity factor (MJ·mm·hm^–2^·h^–1^·a^–1^), and *K* is soil erodibility factor (t·hm^2^·h·MJ^–1^·mm^–1^·hm^–2^). *L* and *S* are dimensionless slope length and slope factors, respectively. *C* is vegetation cover factor, and *P* is soil and water conservation measures factor, respectively. *P* utilized an assignment method, with the following *P* values: 0.15 for paddy field, 0.35 for dry land, 1.00 for forest land, 0.80 for grassland, 0.00 for water area and construction land, and 1.00 for naked land. *R_i_* is rainfall erosivity force of the i-th half-monthly time period. *P_j_* is erosive daily rainfall on the j-th day of the half-month time period (mm). *P_d_*_12_ and *Py*_12_ represent the daily average rainfall for days with at least 12 mm of rain, and the annual average rainfall for those same days, respectively. Where *FVC* is the fractional vegetation cover, *NDVI_max_* and *NDVI_min_* are the *NDVI* values of the areas with complete vegetation cover and no vegetation cover, respectively. For more information on the calculation methods, refer to Shi et al. (2021); Liu (2024); Tang et al. (2023a) and Liu et al. (2022a).

#### 2.3.2 Barycenter migration model

The barycenter migration model can be used to depict the temporal evolution of erosion intensity levels in space, symbolizing the shifting trend of the erosion center’s gravity space, thereby elucidating the dynamic change features and spatial distribution law of the erosion process (Dai et al., 2022; Zhang et al., 2021b). As follows:


(12) $$X_{m}=\frac{\sum_{i=1}^{n}\left(c_{mi}x_{i}\right)}{\sum_{i=1}^{n}c_{mi}},\;Y_{m}=\frac{\sum_{i=1}^{n}\left(c_{mi}y_{i}\right)}{\sum_{i=1}^{n}c_{mi}}$$

where *X_m_* and *Y_m_* denote the latitude and longitude of the center of soil erosion gravity of the spatial distribution in the m-th year, respectively. *C_mi_* refers to the value of erosion intensity for the i-th raster, and *x_i_* and *y_i_* denote the latitude and longitude of the i-th raster.

#### 2.3.3 Geographical detector

This paper primarily uses factor detection, one of four detectors, to analyze the effects of different independent variables on soil erosion (Wang and Xu, 2017; Wang et al., 2022b). Specifically, erosion intensity is the dependent variable, while each influencing factor serves as the independent variable.


(13) $$q=1-\frac{1}{N\sigma^{2}}\sum_{h=1}^{L}N_{h}\sigma_{h}^{2}$$

where *h* is the stratification of the independent variable, *N_h_* and *N*, $\sigma_{\text{h}}^{2}$ and *σ* ^2^ denote the number of cells, variance in stratum *h*, and the whole region, respectively. The *q*-value signifies the explanatory power of the corresponding influence factor on the spatial variability of soil erosion intensity. It ranges from 0 to 1, with a higher value suggesting stronger spatial variability and explanatory power of the dependent variable.

#### 2.3.4 Minimum cumulative resistance model

For the purpose of analyzing the amount of effort a species must expend when migrating from its source to its destination, Knaapen initially suggested the MCR model in 1992 (Sun et al., 2024). The essence of the model is to analyze and find the passage of humans or other organisms to overcome the resistance of the least cumulative landscape element from the point of origin (source) to the destination (sink), which is calculated by the following formula:


(14) $$MCR=f_{\min}\left(\sum_{j=n}^{i=m}D_{ij}\times R_{i}\right)$$

where *MCR* is the MCR value. *D_ij_* and *R_i_* represent the spatial distance from the source (i) to a point (j) and resistance to spatial expansion by source (*i*), respectively. The function *f* represents the distance from any point in space to all sources, and it is monotonically increasing.

In this study, the area with severe erosion intensity is selected as the “source of soil erosion”, and the area spreading out along the “source of soil erosion” is divided into SECA, soil erosion buffer area, soil erosion sensitive area, and soil erosion monitoring area by using MCR model. SECA is geographically closest to the “source of soil erosion”, has relatively weak soil and water conservation capacity, and is highly susceptible to soil erosion problems. The soil erosion buffer area has some soil conservation capacity and can take certain measures to mitigate and reverse the state of soil erosion, serving as buffer zones between SECA and soil erosion sensitive area. The soil erosion sensitive area has better ecological functions, a certain soil retention capacity and risk resistance, but the ecosystem is vulnerable to external interference and thus prone to soil erosion. The ecosystem in soil erosion monitoring area farthest from the “source of soil erosion” is well-functioning with strong soil retention capacity, and most of the area is slightly eroded, so it is sufficient to maintain monitoring of soil erosion intensity to prevent large-scale erosion.

## 3 Results

### 3.1 Spatio-temporal variation characteristics of soil erosion in the JRB

**Figure 2:**
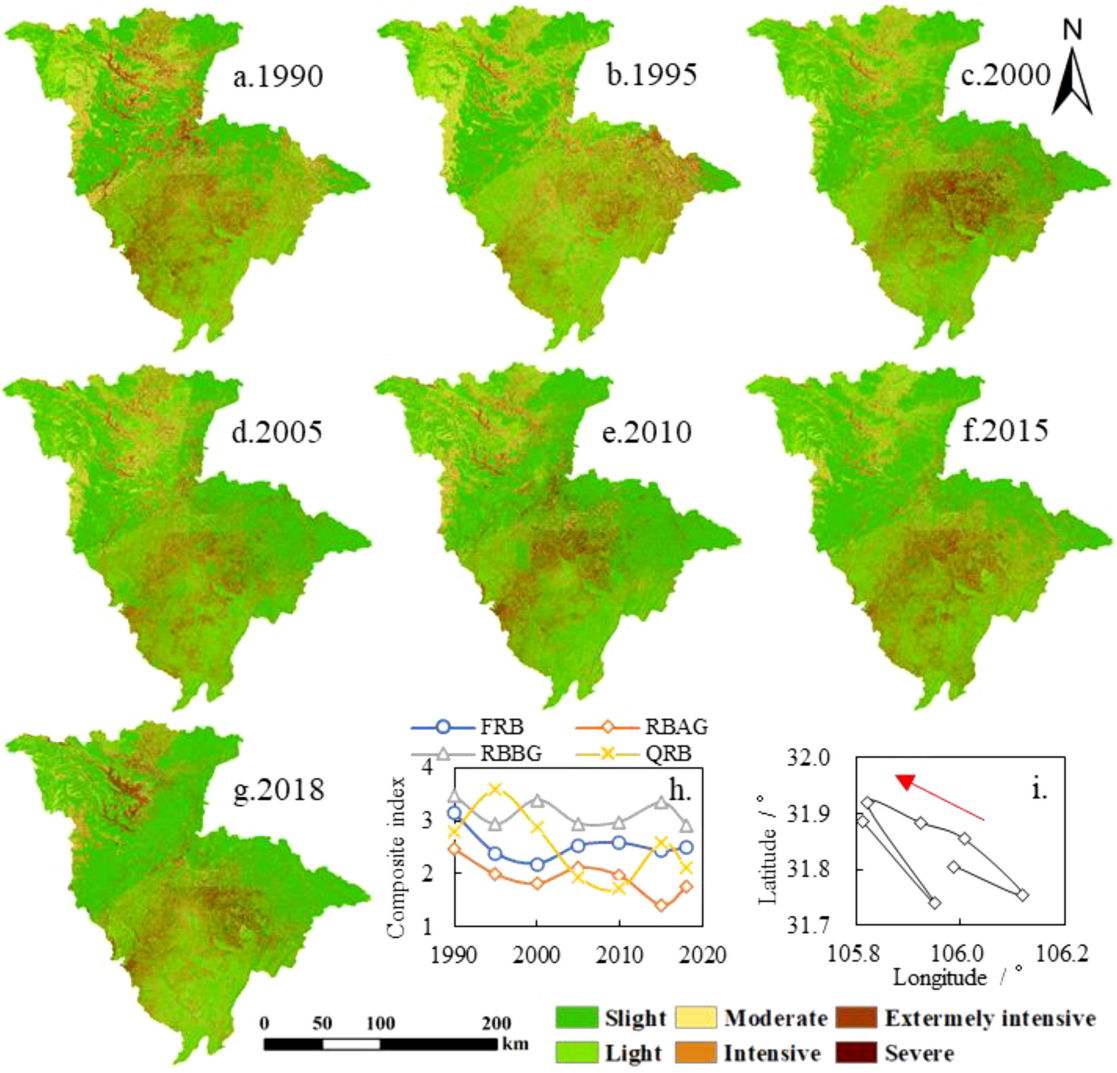
Intensity of soil erosion in JRB from 1990-2018 **(a-g)**, spatial distribution of soil erosion. **(h)**, composite index of soil erosion intensity. **(i)** shift of the center of gravity of soil erosion with moderate or above erosion intensity.

To provide a reference for the development of scientific soil erosion management and related research, this study classified SEM in the JRB from 1990 to 2018 based on the RUSLE model (calculated using Equations 1–11), following the Chinese water conservancy industry standard “Standards for classification and gradation of soil erosion (SL 190-2007)” formulated by the Ministry of Water Resources of China (**Figures 2a–g**). Soil erosion intensity is divided into slight, light, moderate, intensive, extremely intensive and severe, corresponding to erosion intensity levels of<500, 500-2,500, 2,500-5,000, 5,000-8,000, 8,000-15,000, and ≥15,000 t·km^–2^·a^–1^, respectively. To compare the soil erosion status between FRB, RBAG, RBBG, and QRB more intuitively, this study weighted the proportion of different soil erosion intensities in the corresponding basin areas of FRB, RBAG, RBBG, and QRB. The weight scores corresponded to different soil erosion intensities (0, 2, 4, 6, 8, and 10 for slight, light, moderate, intensive, extremely intensive and severe, respectively). The calculated values, called the composite index of soil erosion intensity, are shown in **Figure 2h**. The shift of the center of gravity of soil erosion with moderate or above erosion intensity in the JRB from 1990 to 2018 (calculated using Equation 12) was shown in **Figure 2i**.

**Figure 3:**
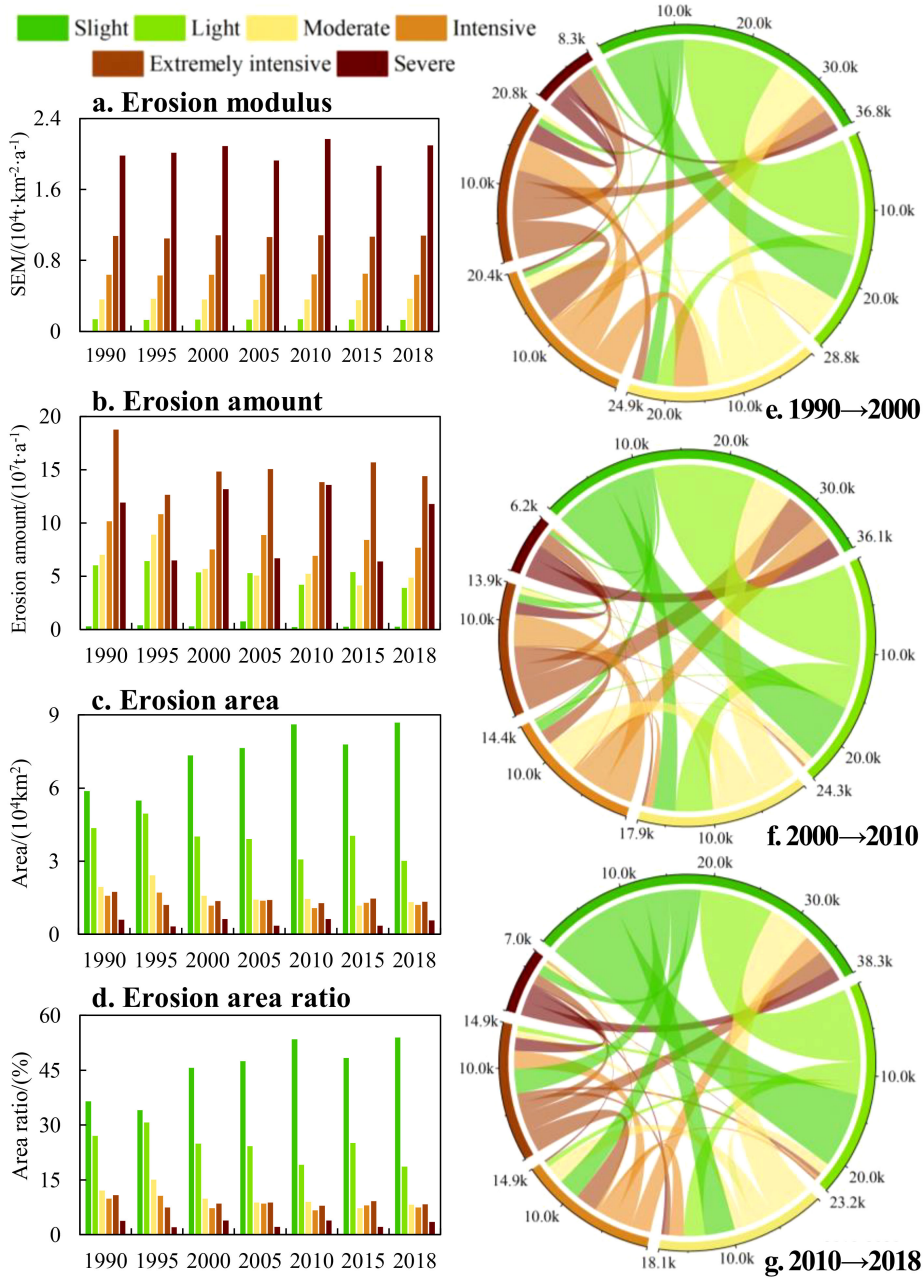
Grade changes in erosion in the JRB from 1990-2018 **(a)**, soil erosion modulus. **(b)**, soil erosion amount. **(c)**, soil erosion area. **(d)**, soil erosion area ratio. **(e-g)** chord diagram of soil erosion transfer matrix from 1990 to 2000, 2000 to 2010, and 2010 to 2018.

The middle and lower JRB, particularly the RBBG, is where the majority of the soil erosion occurs (**Figures 2a–g**). The center of gravity for erosion in JRB is mainly in the RBBG and is more influenced by erosion changes in the QRB (**Figures 2h, i**). The average SEM in the JRB from 1990 to 2018 was 2799 t·km^–2^·a^–1^. According to the intensity level of soil erosion, the mean values of slight, light, moderate, intensive, extremely intensive and severe were 50, 1,339, 3,619, 6,412, 10,727, and 20,202 t·km^–2^·a^–1^, respectively. **Figure 3** and the data from 1990-2018 reveal that erosion intensity was mostly slight and light, accounting for an average of 69.9%. Specifically, slight erosion accounted for 45.6% of the basin area, while light erosion accounted for 24.3%. Moderate, intensive, extremely intensive, and severe intensity only accounted for 10.0%, 8.4%, 8.7%, and 3.0%, respectively. That is, the higher the erosion intensity level, the smaller the corresponding area and proportion. From 1990-2018, the change in SEM corresponding to each grade of erosion intensity was small, and the decrease in erosion amount in the basin mostly came from a decrease in the corresponding area. It can be seen from **Figure 2g** that the erosion in the upper reaches of the JRB in 2018 showed signs of aggravation. This is because in 2018, a catastrophic flood occurred in the JRB due to persistent heavy rainfall, especially in the RBAG. This event also directly promoted the formation of the No.2 flood in the YRB in 2018 (China’s water conservancy department numbered the floods that occurred in the main rivers and reached the prescribed standards every year; the standard of the No.2 flood in the YRB in 2018 is that the flow of the Cuntan hydrological station on the main stream of the YRB, about 7.5 km from the intersection of the YRB and the JRB, rose and exceeded the standard of 50,000 m^3^/s to 50,400 m^3^/s at 4:00 on July 13, 2018). The average SEM of the JRB decreased from 3,332 t·km^–2^·a^–1^ in 1990 to 2,686 t·km^–2^·a^–1^ in 2018, consistent with existing research on SEM in the JRB (Li and Wang, 2024). This also indicates that the once-thriving problem of erosion has been successfully contained and regulated within the basin.

Given the multifaceted nature of soil erosion, influenced by a plethora of interrelated factors, we meticulously selected a total of six key factors - slope aspect, slope, elevation, land use, precipitation, and vegetation (NDVI) - and analyzed their impacts on soil erosion in the JRB using the geographical detector (calculated using Equation 13). **Table 1** presents the results, which revealed that land use contributed the most, amounting to 25.1% in 2015. Vegetation followed, then slope, elevation, and precipitation, while the influence of slope aspect was small to negligible. Consequently, this study emphasizes the effect of land use on soil erosion.

**Table 1:** The results of factor detection in the JRB.

Independent variable	1990	1995	2000	2005	2010	2015	2018
Slope aspect	0.001	0.001	0.002	0.003	0.006	0.003	0.003
Slope	0.098	0.106	0.038	0.052	0.036	0.107	0.046
Elevation	0.047	0.054	0.06	0.032	0.018	0.093	0.039
Land use	0.198	0.165	.205	0.183	0.144	0.252	0.162
Precipitation	0.018	0.053	0.052	0.036	0.017	0.09	0.011
Vegetation	0.154	0.143	0.154	0.142	0.13	0.12	0.251

All *p*-values are less than 0.05.

### 3.2 Analysis of landscape pattern change characteristics in the JRB

**Figure 4:**
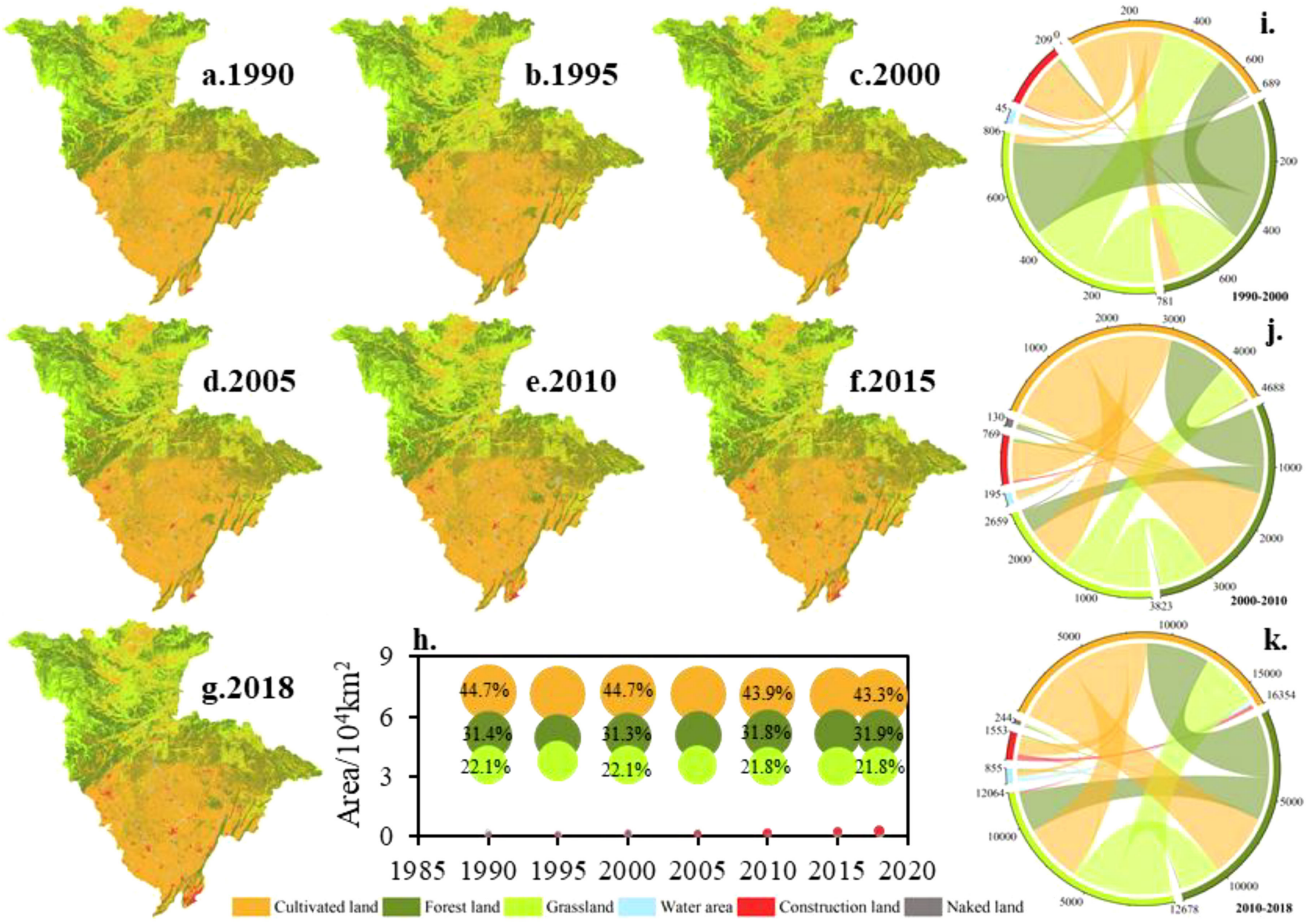
The JRB’s land use changes from 1990 to 2018 **(a-g)**, spatial distribution of land use. **(h)**, area and proportion of each land use type. **(i-k)** chord diagram of land use transfer matrix from 1990 to 2000, 2000 to 2010, and 2010 to 2018.

The JRB is mainly made up of cultivated land, forest land, and grassland, covering 44%, 31%, and 22%, respectively, with a combined area of over 97% (**Figure 4h**). Cultivated land is predominantly found in the middle and lower JRB, while forest land and grassland are alternatively distributed in the middle and upper reaches (**Figures 4a–g**). Construction land mainly aligns with the river system’s distribution. From 1990 to 2018, ecological protection policies were promulgated and implemented continuously, and regional urbanization construction increased, leading to a significant expansion of forest land area, a gradual increase in construction land, and a reduction in cultivated land (**Figures 4i–k**).

**Figure 5:**
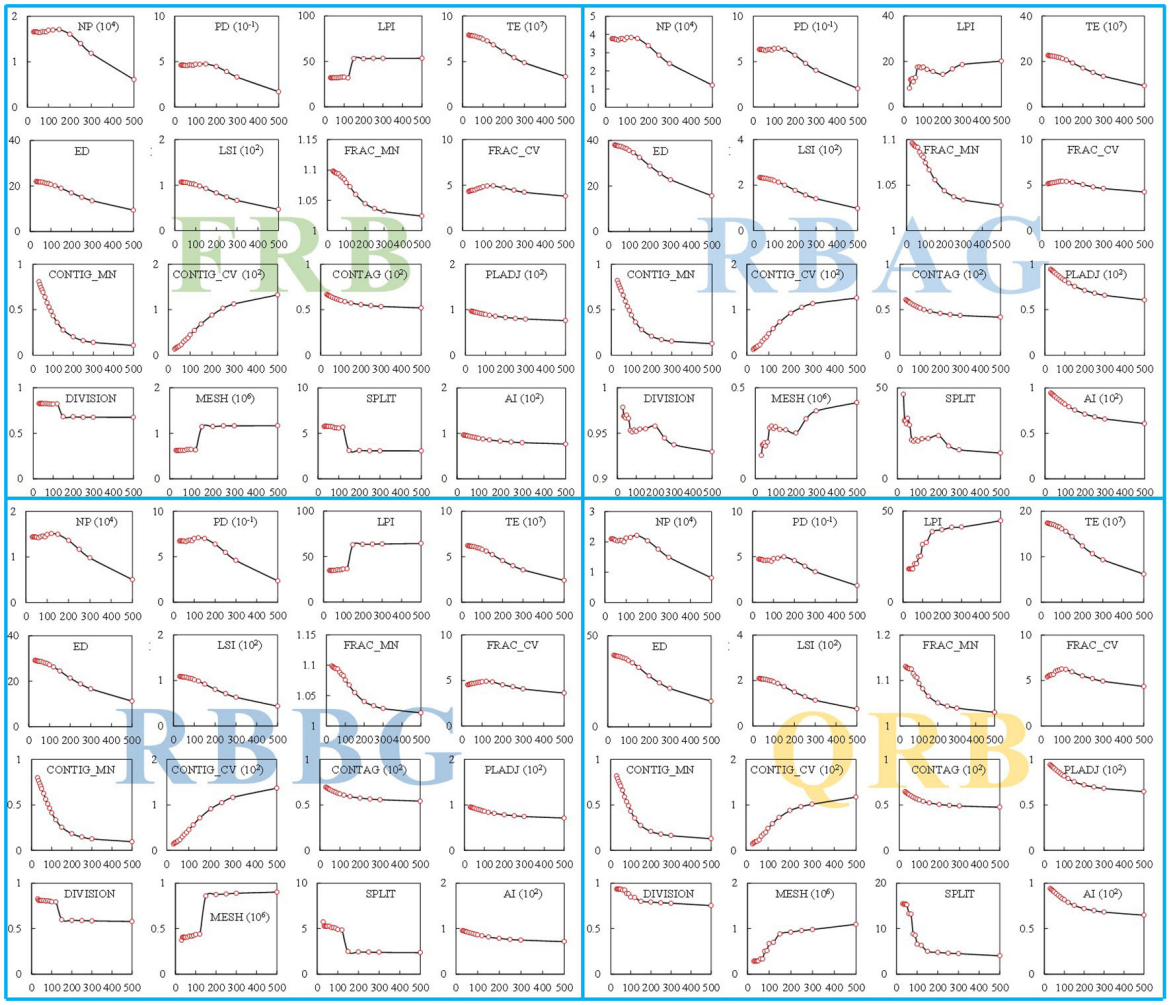
Grain-size effects of landscape patterns in sub-basins of the JRB.

Landscape pattern indices can quantitatively reflect dynamic changes in land use patterns. This study used the Fragstats software to calculate landscape pattern indices. Different indices were selected, and 50 m and 1,000 m were chosen as suitable scales for analyzing the granularity and magnitude of landscape pattern indices, respectively, using methods such as area information conservation evaluation, coefficient of variation, and semi-variance analysis (**Figure 5**).

The appropriate scale analysis of the landscape pattern of the JRB indicated an increase in the number of patches (NP) and patch density (PD) indices over the years, especially after 2005 (**Table 2**). Edge density (ED), landscape shape index (LSI), and average fractal dimension index (FRAC_MN) increased while average contiguity index (CONTIG_MN) and variance of contiguity index (CONTIG_CV) slightly decreased. Contagion (CONTAG), percentage of like adjacencies (PLADJ), and aggregation index (AI) showed a decreasing trend while landscape division index (DIVISION), interspersion and juxtaposition index (IJI), splitting index (SPLIT) showed an increasing trend. Simpson’s diversity index (SIDI), There was a modest rise in the Simpson’s evenness index (SIEI), Shannon’s evenness index (SHEI), and Shannon’s diversity index (SHDI).

**Table 2:** Changes of landscape pattern index in the JRB.

Type	Metric (acronym)	1990	1995	2000	2005	2010	2015	2018
Area-Edge metrics	LPI	9.1406	9.1304	9.1247	9.0951	9.6139	8.8280	8.777
	TE	533247400	532137250	534585400	548286950	545810300	538544750	557056950
	ED	33.0907	33.0223	33.1737	34.0237	33.8697	33.4191	34.5884
Shape metrics	PAFRAC	1.5720	1.5841	1.5782	1.5973	1.5832	1.5696	1.5809
	SHAPE_MN	2.0794	2.1045	2.0858	2.1397	2.0983	2.0580	2.1139
	SHAPE_CV	90.3928	90.3554	90.3938	92.2705	90.6713	89.4431	90.9340
	CIRCLE_MN	0.6670	0.6729	0.6683	0.6718	0.6684	0.6649	0.6679
	CIRCLE_CV	22.0688	21.1353	21.7506	21.3862	21.7152	21.9307	21.9672
	CONTIG_MN	0.6982	0.7042	0.7012	0.7007	0.7013	0.7015	0.6952
	CONTIG_CV	20.4899	18.9681	19.7123	19.152	19.5033	19.9272	20.3668
Aggregation metrics	CONTAG	58.1730	58.0388	58.0110	57.6433	57.2380	57.0726	56.5065
	IJI	49.6401	49.5997	49.8111	49.9656	51.3107	51.9520	52.2374
	PLADJ	91.6968	91.7122	91.6762	91.4639	91.5032	91.6155	91.3018
	AI	91.7187	91.734	91.6981	91.4858	91.5254	91.6378	91.3242
	LSI	334.5234	333.9744	335.3469	343.865	342.2561	337.7597	351.1112
	COHESION	99.8535	99.8449	99.8521	99.8543	99.8565	99.8515	99.8468
	NP	87912	86099	87543	85619	88780	90790	91056
	PD	0.5455	0.5343	0.5432	0.5313	0.5509	0.5634	0.5654
	DIVISION	0.9681	0.9699	0.9684	0.9684	0.9674	0.9691	0.9713
	SPLIT	31.321	33.2112	31.6128	31.6319	30.6965	32.3596	34.8219
	MESH	514503.5	485211.66	509753.85	509449.8	524977.71	497993.54	462505.39
	CONNECT	0.0199	0.0201	0.0199	0.0203	0.0197	0.0194	0.0195
Diversity metrics	SHDI	1.1479	1.1533	1.1528	1.1589	1.1729	1.1816	1.1919
	SIDI	0.6527	0.6555	0.6533	0.6551	0.6581	0.6601	0.6627
	SHEI	0.6407	0.6437	0.6434	0.6468	0.6546	0.6595	0.6652
	SIEI	0.7832	0.7865	0.7840	0.7861	0.7897	0.7922	0.7953

This indicates that since 1990, the indices within the basin have been characterized by increasing complexity, increasing fragmentation, and decreasing connectivity between landscape patches and becoming more dispersed, with 2005 being the main node of landscape pattern change. The reason for this may be that the gradual implementation of the ecological management project has led to some changes in cultivated land and forest and grassland, resulting in the migration of material and energy between patches within the landscape, which has increased the emergence of small-sized landscape patches and increased the complexity of the landscape within the study area.

### 3.3 Analysis of the effect of soil erosion resulting from landscape patterns

**Figure 6:**
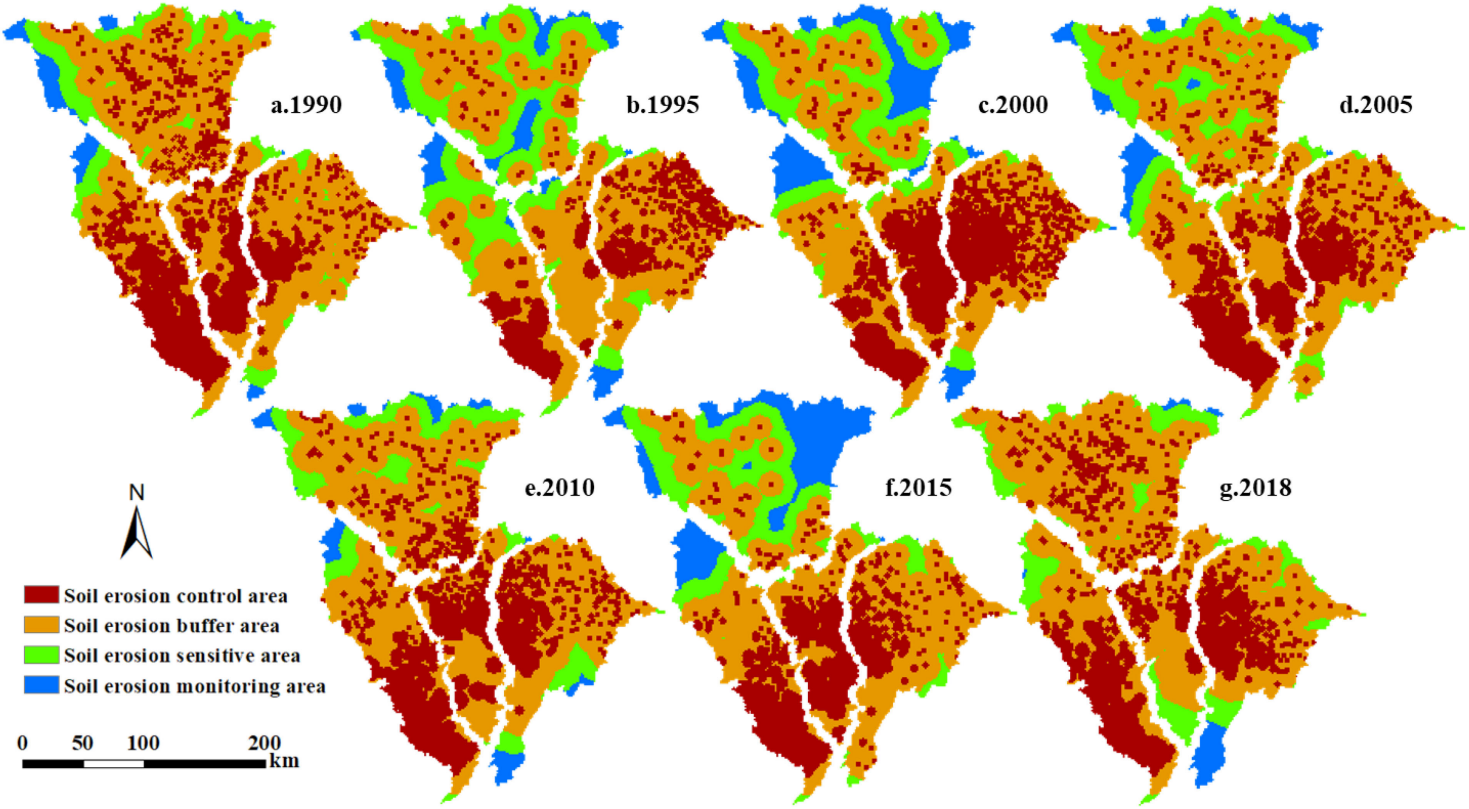
**(a-g)** Division of areas for mitigating soil erosion in the JRB from 1990-2018.

The JRB was divided into 110 sub-basins, of which the FRB, RBAG, RBBG, and QRB contain 22, 37, 17, and 34 sub-basins, respectively. From 1990-2018, the SEM of each sub-basin was treated as the dependent variable, and the landscape patterns of the corresponding year were considered the independent variables. The correlation analysis, shown in **Table 3**, revealed that the effect of some indices was not significant. After comprehensive consideration, TE, CONTIG_CV, AI, NP, MESH, and CONNECT were excluded. In addition, it was found that landscape pattern indices such as PD, which characterize the degree of landscape fragmentation, were significantly positively correlated with SEM (*p*< 0.05), indicating that higher patch density means more edges and less continuous vegetation cover, which may increase erosion. The diversity indexes such as SHDI and SIDI are negatively correlated with SEM, which indicates that diversified landscapes may contain more types of soil conservation functions, thereby reducing the possibility of erosion. Next, the partial least squares regression method was used to obtain 10 landscape pattern indices with strong explanatory power for SEM. Then, the severe erosion area was selected as the “source of soil erosion”, and the MCR model (calculated using Equation 14) was utilized to categorize the areas spreading outward along the “source of soil erosion” into four regions: SECA, soil erosion buffer area, soil erosion sensitive area and soil erosion monitoring area (**Figure 6**).

**Table 3:** SEM and landscape pattern correlation analysis.

Type	Metric (acronym)	1990	1995	2000	2005	2010	2015	2018
Area-Edge metrics	LPI	0.1240	0.0800	0.1830	0.1660	0.1410	0.242*	0.246**
	TE	-0.1070	-0.0550	-0.215*	-0.244*	-0.1750	-0.334**	-0.324**
	ED	0.1330	0.297**	0.1110	0.0170	0.0120	-0.0340	-0.0960
Shape metrics	PAFRAC	0.0440	0.215*	0.1170	0.0320	-0.0130	-0.0280	-0.1870
	SHAPE_MN	-0.272**	0.1760	-0.0320	-0.371**	-0.188*	-0.247**	-0.274**
	SHAPE_CV	-0.280**	-0.1010	-0.286**	-0.389**	-0.345**	-0.467**	-0.342**
	CIRCLE_MN	0.1340	0.1090	0.223*	0.215*	0.303**	0.308**	0.1110
	CIRCLE_CV	-0.214*	0.1230	-0.0550	-0.300**	-0.299**	-0.1790	-0.0330
	CONTIG_MN	-0.1070	-0.355**	-0.223*	-0.0390	0.0910	-0.233*	-0.1760
	CONTIG_CV	-0.198*	0.203*	-0.0250	-0.287**	-0.246**	-0.1180	-0.0070
Aggregation metrics	CONTAG	0.1110	0.0290	0.225*	0.211*	0.0710	0.1820	0.1290
	IJI	-0.0610	-0.305**	-0.208*	0.0360	0.0890	0.0410	0.1000
	PLADJ	-0.1370	-0.302**	-0.1310	-0.0300	-0.0310	0.0140	0.0930
	AI	-0.1370	-0.302**	-0.1230	-0.0220	-0.0140	0.0270	0.0960
	LSI	-0.0480	0.0500	-0.1340	-0.196*	-0.1390	-0.282**	-0.293**
	COHESION	0.0210	-0.0350	0.0140	-0.0220	-0.0190	0.0180	0.0520
	NP	0.1530	-0.0160	-0.0610	0.0620	-0.0050	-0.1070	-0.1410
	PD	0.485**	0.210*	0.271**	0.468**	0.335**	0.319**	0.233*
	DIVISION	-0.1560	-0.0820	-0.227*	-0.234*	-0.1780	-0.316**	-0.288**
	SPLIT	-0.1560	-0.0820	-0.227*	-0.234*	-0.1780	-0.316**	-0.288**
	MESH	-0.0010	-0.0890	-0.0210	-0.0400	-0.0030	0.0080	0.0340
	CONNECT	-0.211*	-0.0440	-0.0450	-0.1420	-0.0760	-0.0080	0.0720
Diversity metrics	SHDI	-0.1260	-0.1390	-0.277**	-0.210*	-0.0480	-0.342**	-0.211*
	SIDI	-0.1870	-0.1360	-0.329**	-0.308**	-0.1460	-0.433**	-0.324**
	SHEI	-0.192*	-0.1770	-0.325**	-0.272**	-0.0890	-0.253**	-0.1350
	SIEI	-0.206*	-0.1620	-0.358**	-0.329**	-0.1630	-0.411**	-0.307**

*represents a significance level of *p*< 0.05, and **represents a significance level of *p*< 0.01.

The results indicated that the SECA (shown in red in **Figure 6**) was predominantly located in the middle and lower JRB, specifically the middle and lower FRB, middle and lower RBBG, and central and western QRB.

When viewed alongside **Figure 4**, it becomes evident that the majority of the cultivated land distribution regions align with the primary distribution areas of SECA. The soil erosion sensitive area (green in **Figure 6**) and soil erosion monitoring area (blue in **Figure 6**) are primarily located in the upper JRB. Combined with land use and DEM, it can be found that forest and grass occupy the main position and the altitude is high in the corresponding area. The soil erosion buffer area (orange in **Figure 6**), situated between the SECA (red in **Figure 6**) and the soil erosion sensitive area (green in **Figure 6**), occupies a relatively large proportion. As a region near the “source of soil erosion”, it can construct a transition zone by limiting the continuous outward diffusion of erosion, thereby delaying soil erosion. From 1990 to 2018, the inter-annual variance of each control zone’s area in the JRB was quite different. Among them, the SECA (red in **Figure 6**) in the RBAG and RBBG showed a decrease, while the SECA (red in **Figure 6**) in the FRB and the QRB fluctuated greatly and showed a phenomenon of concentration to the downstream and the central and western regions, respectively.

## 4 Discussion

### 4.1 Changes in soil erosion and the factors that influence it in the UYR

Soil erosion analysis in the JRB located in the UYR from 1990 to 2018 revealed a decrease in the SEM. This was due to the 1988 approval of the UYR as a national key prevention and control area for soil and water conservation by the Chinese government (known locally as the “Changzhi” project), followed by the implementation of the “Changzhi” project in the middle and lower JRB in the UYR the next year (Jiang et al., 2024). In 2013, the Chinese Ministry of Water Resources announced that the upper JRB was the primary region for preventing soil erosion, while the middle and lower JRB remained the key areas for control. From the variation of SEM in each sub-basin of JRB (**Figure 2h**), it can be found that the fluctuation range of SEM in QRB is relatively large, which also leads to the shift of erosion center of gravity in the JRB. This could be due to QRB being geographically closer to the fan-shaped basin than FRB, RBAG, and RBBG. Additionally, the upstream region has a larger area relative to the downstream area and is situated in the Daba Mountain rainstorm area (Qi et al., 2022; Wang et al., 2021). Combined with the unique weather system of the region, the southwest vortex (Xiao et al., 2024), this results in floods from multiple parallel tributaries in the upstream, converging into larger floods in the lower reaches. This, in turn, leads to serious water and soil loss in the lower reaches where the terrain is mostly shallow hill and flatland, and cultivated land is concentrated.

This study’s findings align with global research, as highlighted by Dethier et al. (2022), which asserts that land use has the most substantial impact on soil erosion during the study period. While some studies have established that climate change is the dominant factor in hydrological changes, human activities remain the dominant factor affecting water and sediment changes on a regional scale for study periods shorter than geological time (Song et al., 2023). The study reveals that erosion remains a significant issue, particularly in the middle and lower JRB, where cultivated land is the primary cause. The problem persists globally, as evidenced by Quinton and Fiener (2024).

It’s important to recognize that changes in land use are only one facet of human activity. Research by Yu et al. (2021) in the YRB found that the most important factor affecting regional soil erosion was economic growth. As China’s most economically dynamic region, the degree of economic growth and the standard of life of the YRB’s citizens will likewise have an impact on soil erosion. Especially in areas with severe erosion, economic growth will make local areas no longer rely on industries with large resource consumption and strong ecosystem interference (such as agriculture). Furthermore, in terms of research methodologies, the partial least squares-structural equation model (PLS-SEM) has been extensively employed in research approaches to analyze erosion and sediment yield drivers (Tian et al., 2022). Therefore, socio-economic factors representing human activities will be introduced in the follow-up of this study, and the results of erosion influencing factors between different methods (such as geographic detector, PLS-SEM) will be compared and analyzed.

### 4.2 Soil erosion in response to landscape pattern in the UYR

The JRB is primarily comprised of cultivated land, forest land, and grassland. The major changes are a decrease in cultivated land and increases in forest land and construction land. This aligns with previous research on the YRB (Yuan et al., 2023), potentially due to the government’s continuous implementation of ecological governance policies and measures in the UYR and recent urbanization (Luo et al., 2023; Wei et al., 2024). The landscape pattern indices have also changed (**Table 2**), showing diversification, complexity, and fragmentation. The correlation between the indices and soil erosion (**Table 3**) suggests that an increase in diversity indices reduces erosion, while an increase in landscape fragmentation indices increases the possibility of erosion, consistent with similar studies (Chen et al., 2023a; Zhang et al., 2017).

The diversity indices are significantly affected by habitats (Yan et al., 2024), while multi-habitats can reduce robust variability at the landscape level and support better ecosystem services (Hackett et al., 2024). By analyzing land use changes in the JRB (**Figure 4**), it is evident that cultivated land conversion to forests mostly occurs in regions with higher altitudes or steeper slopes, while the increase in construction land is mainly occupied by cultivated land at low-altitude or with gentle slopes. This is also basically consistent with land use changes throughout China (Liu et al., 2023a). The Chinese government has given a greater emphasis on food security in recent years since recognizing it as crucial for the nation’s stability and social harmony. This has resulted in the implementation of numerous strict policies, demonstrating the government’s commitment to ensuring the long-term sustainability of cultivated land. In particular, these policies limit the occupation of cultivated land by construction land. However, the development of infrastructure still encroaches on scattered cultivated lands. The relatively small increase in construction land area may seem insignificant, but it significantly increases impervious surfaces and fragments the agricultural landscape, which is already highly impacted by human activities. This, in turn, enhances the basin’s ability to generate runoff and sediment production and transport, while also increasing sediment connectivity.

This implies that in our thorough assessment of the effects of returning previously cultivated land back to its natural state or forest and grassland on soil erosion levels, we should not simply limit our considerations to the quantifiable change in the restored land area. Instead, we must also take into account and give due importance to the far-reaching implications of the transformation at the landscape level. The results of erosion zoning (**Figure 5**) indicate that the middle and lower JRB remain the primary and most critical areas for rigorous soil erosion control measures. Under the restriction of the cultivated land minimum of 1.8 billion mu (Li and Song, 2023), in order to reduce erosion risk, different measures can be implemented by improving the concentration and connectivity of the landscape in the basin. For instance, in the SECA, some sloping cultivated land should be transformed into terraces, and scattered and contracted cultivated land should be integrated into centralized contracted management to avoid fragmented cultivated land and additional increased agricultural infrastructure, scattered rural residents will be relocated and centralized resettled, low impact development and sponge city construction will be closely combined for construction land, and vegetation buffer zones will be added to impermeable areas.

## 5 Conclusions

This study analyzed the spatio-temporal variations of SEM in JRB from 1990-2018, a typical basin in the UYR. And the geographical detector was used to obtain that land use had a comparatively larger impact on soil erosion during this period. The land use and landscape indices of the basin, as well as their relationship with SEM, were then analyzed. The MCR model was used to categorize erosion control zones in the basin.

1. Soil erosion intensity from 1990 to 2018 was mainly slight and light, with the middle and lower JRB having the highest concentrations of moderate and higher intensity. The average SEM decreased from 3,332 to 2686 t·km^–2^·a^–1^. The biggest factor influencing basin erosion was land use.
2. Land use is primarily cultivated land, forest, and grassland, comprising 44%, 31%, and 22%, respectively, and the cultivated land is mostly found in the middle and lower JRB. From 1990 to 2018, the inter-class changes showed an expansion of forest and construction land and a shrinkage of cultivated land. The basin’s overall landscape pattern became more complex, fragmented, and less connected between landscape patches, tending towards dispersion.
3. The SECA is primarily located in the middle and lower JRB and features predominantly cultivated land use. From 1990 to 2018, the SECA underwent significant area changes. Specifically, the SECA in the RBAG and RBBG decreased, while the SECA in the QRB and FRB fluctuated greatly and showed a concentration in the central-western and downstream areas, respectively.

## Data Availability

The original contributions presented in the study are included in the article/supplementary material. Further inquiries can be directed to the corresponding author.
